# Elevated tissue inhibitor of metalloproteinase-1 along with left atrium hypertrophy predict atrial fibrillation recurrence after catheter ablation

**DOI:** 10.3389/fcvm.2022.1010443

**Published:** 2022-11-01

**Authors:** Weiping Sun, Haiwei Li, Zefeng Wang, Qin Li, Haichu Wen, Yongquan Wu, Jie Du

**Affiliations:** ^1^Department of Cardiology, Beijing Anzhen Hospital, Capital Medical University, Beijing, China; ^2^Beijing Institute of Heart Lung and Blood Vessel Disease, Beijing, China

**Keywords:** atrial fibrillation, biomarkers, TIMP metallopeptidase inhibitor 1, recurrence, left atrium diameter

## Abstract

This study aimed to establish a model that predicts atrial fibrillation (AF) recurrence after catheter ablation using clinical risk factors and biomarkers. We used a prospective cohort study, including 230 consecutive persistent AF patients successfully treated with catheter ablation from January 2019 to December 2020 in our hospital. AF recurrence was followed-up after catheter ablation, and clinical risk factors and biomarkers for AF recurrence were analyzed. AF recurred after radiofrequency ablation in 72 (31%) patients. Multiple multivariate logistic regression analysis demonstrated that tissue inhibitor of metalloproteinase-1 (TIMP-1) and left atrium diameter (LAd) were closely associated with AF recurrence. The prediction model constructed by combining TIMP-1 and LAd effectively predicted AF recurrence. Additionally, the model’s performance discrimination, accuracy, and calibration were confirmed through internal validation using bootstrap resampling (1,000 times). The model showed good fitting (Hosmer–Lemeshow goodness chi-square 3.76138, *p* = 0.926) and had a superior discrimination ability (the area under the receiver operation characteristic curve0.917; 95% CI 0.882–0.952). The calibration curve showed good agreement between the predicted probability and the actual probability. Moreover, the decision curve analysis (DCA) showed the clinical useful of the nomogram. In conclusion, our predictive model based on serum TIMP-1 and LAd levels could predict AF recurrence after catheter ablation.

## Introduction

Atrial fibrillation (AF) is the most common arrhythmia. The prevalence of AF in adults has been reported to be between 2 and 4%, and the annual rate of paroxysmal AF progression to persistent AF ranges from < 1 to 15% (1, 2). Furthermore, AF is a major risk factor for stroke and heart failure (3).

Catheter ablation has become the routine treatment for AF. However, it is associated with a low success rate and high recurrence rate (4); patients experiencing AF recurrence require repeat procedures. Owing to possible recurrence in up to 50% cases, patient selection is crucial for optimal catheter ablation as shown in the STAR AF II trial (5). Currently, the most powerful and independent preprocedural predictor of AF recurrence after radiofrequency (RF) ablation is left atrium (LA) diameter (LAd) (6). Moreover, atrial remodeling leads to atrial dilatation, which reduces the effectiveness of radiofrequency ablation for AF (7). LAd is associated with AF recurrence after catheter ablation and is an independent predictor of AF recurrence (7). However, the clinical value of LA size in selecting patients for catheter ablation is limited. Severe enlargement of the LA can predict AF recurrence, but mild-to-moderate enlargement of the LA has a mixed response to RF catheter ablation. However, the relationship between LA size and atrial fibrosis is uncertain (7). Therefore, LA size alone is not enough to predict AF recurrence following RF ablation. Clinically, the main method used to evaluate the degree of atrial fibrosis is contrast-enhanced MRI (CE-MRI). However, CE-MRI is not widely available, and for patients with severe renal dysfunction, its use of contrast may not be recommended. Circulatory fibrosis markers have the potential to replace MRI. Including atrial fibrosis biomarkers associated with AF in risk models may yield more precisive predictions of AF risk. Previous studies have shown that tissue inhibitor of metalloproteinase-1 (TIMP-1) levels in the atrial appendage tissue, which is correlated with left atrial diameter, are higher in patients with AF than in patients with sinus rhythm (8, 9). Moreover, TIMP-1 promotes myocardial fibrosis by mediating CD63-integrin β1 interactions (10). AF progression is associated with a gradual increase in the expression of matrix metalloproteinase 9 (MMP9)/TIMP-1 (11). The activities of MMP9 that can degrade matrix might be expected to be under-expressed in fibrosis. MMP9 can modulate arrange of biological process. TIMP-1 is an inhibitory molecule that regulates matrix metalloproteinases and plays a crucial role in extracellular matrix. An imbalance of activity between TIMPs and MMPs can lead to the degeneration and replacement of the extracellular matrix, leading to atrial remodeling and fibrosis. Fibrosis can lead to left atrium enlargement and lead to electrical remodeling and structure remodeling in AF (12), which is the mechanism of maintaining persistent AF.

In this study, we tested a hypothesis that changes in structure and remodeling-related biomarkers could predict AF recurrence after RF catheter ablation.

## Materials and methods

### Participants

Data of patients with AF undergoing catheter ablation were retrieved from the medical records of our hospital from January 1, 2019 to December 30, 2020. Patients were enrolled if they met the following inclusion criteria: age > 18 years and a diagnosis of AF based on the 2020 ESC AF guidelines made by two expert cardiologists (3). Persistent AF was determined using 12-lead electrocardiography and/or 24 h Holter monitoring as lasting for more than 7 days. AF recurrence was defined as the existence of AF confirmed by 12-lead electrocardiography and Holter electrocardiography 3 months after RF ablation (13). Exclusion criteria included presence of other concomitant cardiac conditions, a malignant tumor, inflammatory response, or end-stage disease. Approval for this study was obtained from the Ethics Committee of our Hospital (No. 2022042X). Written informed consent were obtained from all patients prior to enrollment.

### Data collection

Clinical and laboratory data were extracted from the medical records by two independent doctors. The clinical data of the patients were collected, including age, sex, hypertension, coronary artery disease (CAD), diabetes mellitus (DM), heart failure (HF), hypertension (HTN), and other indicators. At the same time, laboratory examinations of the patients were conducted, including white blood cell, red blood cell, and platelet counts, and hemoglobin levels. Left atrium diameter (LAd), left ventricular end of systolic diameter (LVESD), left ventricular end of diastolic diameter (LVEDD), and left ventricular ejection fraction (LVEF) were measured. Blood levels of TIMP-1, high-sensitivity C-reactive protein (hs-CRP), and B-type natriuretic peptide (BNP) were measured. The CHA_2_DS_2_-VASc and HAS-BLED scores were calculated for all the patients. AF recurrence was followed up by telephone after AF ablation.

### Statistical analysis

Continuous data were expressed as mean ± standard deviation and analyzed using Student’s *t*-test (comparisons between two groups). Non-normally distributed continuous data were described as median and interquartile range and compared between groups using the Kolmogorov–Smirnov test. Categorical variables are presented as numbers (percentages) and were analyzed using the chi-square test or Fisher’s exact test. Parameters with values of *p* < 0.1 in univariable logistic regression analysis were included in multivariate logistic regression analysis using the backward LR method. The final model was selected based on the AIC rule. Differentiating the diagnosis degree as determined by area under curve (AUC) and Hosmer–Lemeshow, calibration analysis, decision curve analysis, and nomograms were analyzed using R software. The receiver operating characteristic (ROC) curve was cross-validated ten times using Stata 15.0. Statistical significance was set at *p* < 0.05. The analyses followed the framework proposed by Steyerberg and Vergouwe for the derivation and validation of the prediction models. The model was internally validated using 1000 bootstrap samples. The final models were presented as nomograms. Clinical usefulness and net benefit were estimated using decision curve analysis. Data were analyzed using R version 3.2, SPSS 23 (IBM Corp., Armonk, NY, USA), and Stata 15.0 (StataCorp.).

## Results

### Baseline characteristics

**FIGURE 1:**
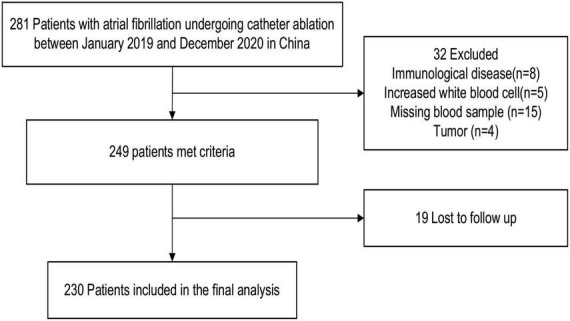
Patient selection flow chart.

Overall, 281 patients admitted to Anzhen Hospital, Capital Medical University, who underwent catheter radiofrequency ablation from January 2019 to December 2020 were reviewed in this study. Figure 1 illustrates the enrollment process of the study participants. Finally, 230 patients with persistent AF were enrolled as 32 patients were excluded for various reasons: 8 patients, autoimmune disease; 4 patients, tumor; 5 patients, elevated white blood cells; and 15 patients, unavailability of blood samples. Furthermore, 19 patients were lost during follow-up. During the follow-up, 72 patients (31%) experienced AF recurrence.

The baseline characteristics are shown in Table 1. Age and sex did not differ significantly between patients with and without recurrence (63.0 [55.0–66.8] vs. 61.0 [51.0–67.0], *p* = 0.315; 48 [66.67] vs. 108 [68.35], *p* = 0.799, respectively). Obesity had significant effect on AF recurrence (BMI: 26.4 [25.1–29.7] vs. 25.4 [23.4–28.4], *p* < 0.05). Patients’ medical history revealed no significant differences between the AF recurrence and no recurrence groups according to CAD, HTN, DM, HF, smoking, or drinking. With regard to laboratory findings, hsCRP and BNP levels were significantly higher in the AF recurrence group than in the no recurrence group (1.5 [0.7–4.1] vs. 1.0 [0.5–2.0], *p* < 0.05, 127.5 [85.0–244.0] vs. 83.5 [44.5–149.0], *p* < 0.001, respectively). There were no significant differences between the AF recurrence and no recurrence groups with regards to estimated glomerular filtration rate (93.0 [80.6–100.4] vs. 94 [85–102.5], *p* = 0.342]. Echocardiographic data including LVEF (62.0 [56.0–65.0] vs. 62.0 [59.0–66.0], *p* = 0.224), LVEDD (48.0 [45.0–52.0] vs. 48.0 [45.0–50.0], *p* = 0.124), and LVESD (31.0 [28.0–35.0] vs. 31.0 [28.0–33.5], *p* = 0.850) between the two groups. The LAd of the AF recurrence group was larger than that of the no recurrence group, and there was a significant difference between the two groups (46.0 [43.0–49.0] vs. 39.0 [36.0–42.0], *p* < 0.001).

**TABLE 1:** Baseline characteristics.

Characteristic	Without recurrence (*n* = 158)	Recurrence (*n* = 72)	*P-*value
Age, years	59.2 ± 10.8	60.7 ± 9.6	0.297
Male (n,%)	108 (68.35)	48 (66.67)	0.799
BMI	25.5 (23.4–28.4)	26.7 (25.1–29.6)	<0.05*
CAD	16 (10.13)	7 (9.72)	0.924
HTN	85 (53.80)	39 (54.17)	0.958
DM	22 (13.92)	7 (9.72)	0.373
HF	6 (3.80)	5 (6.94)	0.305
Smoking	45 (28.48)	20 (27.78)	0.913
Drinking	11 (6.96)	4 (5.56)	0.689
CHA_2_DS_2_-VASc (n,%)			0.231
0 or 1	94 (59.49)	39 (54.16)	
2 or 3	52 (32.91)	26 (36.11)	
≥4	12 (7.6)	7 (9.72)	
LVEF,%	62.0 (59.0–66.0)	62.0 (56.5–65.0)	0.224
LVEDD, mm	47.5 ± 4.2	48.6 ± 4.6	0.078
LVESD, mm	31.0 (28.0–33.0)	31.0 (28.0–35.0)	0.893
eGFR	94.6 (85.0–102.4)	93.5 (80.6–100.2)	0.342
hs-CRP, mg/l	1.0 (0.5–2.0)	1.2 (0.7–3.8)	<0.05*
BNP, pg/ml	94.0 (49.0–142.0)	115.0 (87.5–233.5)	<0.01**
LAd, mm	39.0 ± 4.5	45.8 ± 4.6	<0.01**
TIMP-1, ng/ml	97.4 (73.3–124.2)	147.3 (87.1–200.1)	<0.01**

BMI, body mass index; CAD, coronary artery disease; HTN, hypertension; DM, diabetes mellitus; HF, heart failure; LVEF, left ventricular ejection fraction; LVEDD, left ventricular end-diastolic dimension; LVESD, left ventricular end-systolic dimension; eGFR, estimated glomerular filtration rate; hs-CRP, high-sensitivity C-reactive protein; BNP, B-type natriuretic peptide; LAd, left atrium diameter; TIMP-1, tissue inhibitor of metalloproteinase-1. *P < 0.05; **P < 0.01.

The concentration of TIMP-1 was significantly higher in the AF recurrence group than in the no recurrence group (147.4 [87.1–200.8] vs. 94.4 [72.4–127.2], *p* < 0.001).

### Development and internal validation of predicting model associated with atrial fibrillation recurrence after catheter ablation

**FIGURE 2:**
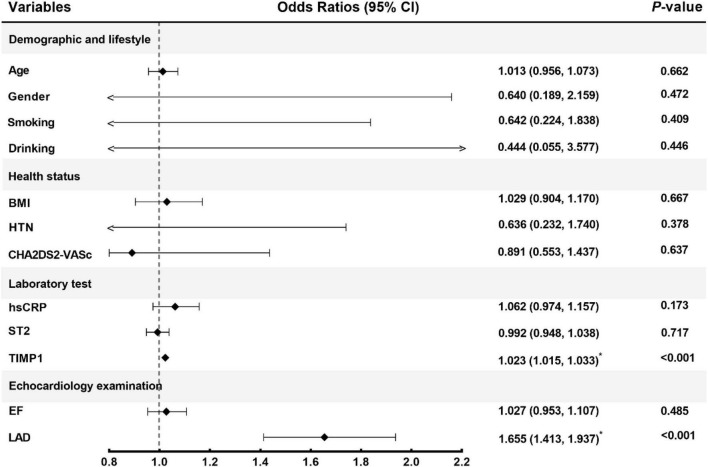
Corrected confounding factors. CI, confidence interval; BMI, body mass index; HTN, hypertension; hsCRP, high-sensitivity C-reactive protein; ST2, Soluble suppression of tumorigenicity-2; TIMP1, tissue inhibitor of metalloproteinase-1; EF, ejection fraction; LAD, left atrium diameter.

**FIGURE 3:**
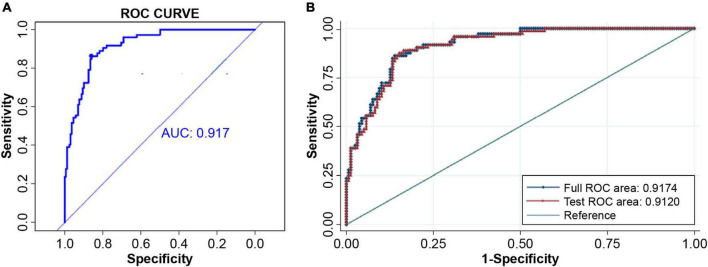
Model discrimination. **(A)** The ROC curve and AUC of the predicting model, 0.917 (95% CI 0.882–0.952). **(B)** The stability of the predicting model discrimination was verified. Cross-validation was done ten times and the area under the ROC curve of the 10-fold cross-validation was AUC = 0.9120. The model discrimination ability was very good. ROC, receiver operating characteristic; AUC, area under curve.

A predictive model for AF recurrence was constructed based on 230 enrolled persistent AF patients and 72 AF recurrences. The predictive factors of the model were selected using univariate binary logistic regression analysis. Variables in the model included clinical risk factors (age, sex, smoking, alcohol consumption, BNP, LVEDD, LVEF, body weight, LAd, hsCRP, and other clinical indicators) and TIMP-1 (Figure 2). The variables included in the model were LAd and TIMP-1 (*p* < 0.05), as determined by multivariate logistic stepwise regression analysis (Table 2). The area under the ROC curve of the model was 0.917 (95% CI, 0.882–0.952). To verify the stability of model discrimination, cross-validation was performed ten times on the model, and the area under the ROC curve, 0.912 (0.861–0.948), revealed a good model discrimination effect (Figure 3).

**TABLE 2:** AF recurrence after radiofrequency ablation: Predictors.

	*B*	OR	95% CI	*P*-value
TIMP-1	0.022	1.023	1.014–1.031	<0.001
LAd	0.434	1.543	1.358–1.752	<0.001
Constant	–21.949			

TIMP-1, tissue inhibitor of metalloproteinase-1; LAd, left atrium diameter.

**FIGURE 4:**
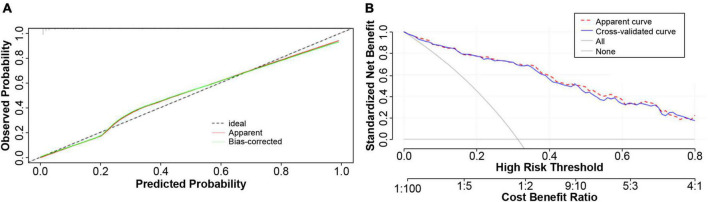
Calibration plot of the predicting model. **(A)** The constructed model was subjected to the degree of calibration Hosmer–Lemeshow (HL) test, and the HL chi-square value was obtained as 3.76138, *P* = 0.926. The results revealed that the prediction accuracy of the model was good. In order to verify the accuracy of the model, the Bootstrap self-help sampling method was conducted 1,000 times to verify the internal accuracy; this indicated that the actual value of the model was in good agreement with the predicted value. **(B)** The model had good clinical net benefit over the probability threshold of 0.5–0.8.

**FIGURE 5:**
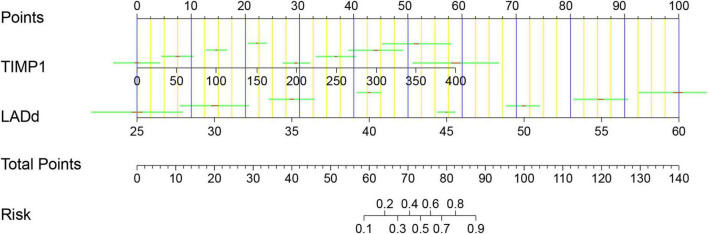
Nomogram for AF recurrence after catheter ablation. Points were assigned for TIMP1 and LAd on the 0–100 scale at the top of the nomogram and may be summed. The number on the “Total Points” scale indicates the corresponding risk predictions of AF recurrence presented beneath. TIMP1, tissue inhibitor of metalloproteinase-1; LAd, left atrium diameter; AF, atrial fibrillation.

In addition, the model calibration ability was tested using the Hosmer–Lemeshow test; The chi-square value was 3.76 (*p* = 0.926), indicating good model prediction accuracy. The bootstrap self-sampling method was used to verify the internal accuracy of the model, and the number of internal samplings was 1,000, indicating that the actual value of the model was in good agreement with the predicted value. The analysis and evaluation of clinical benefits by the decision curve revealed that the prediction model had good clinical benefits (Figure 4). Clinical implementation of the prediction model was based on the nomogram (Figure 5).

## Discussion

We present a novel model to predict AF recurrence after catheter ablation, based on a combination of LAd and fibrosis biomarker TIMP-1. The model showed good fitting and superior discrimination ability. The calibration curve shows agreement between the predicted probability and the actual probability. Moreover, the decision curve analysis showed the clinical utility of the nomogram. Overall, the model has clinical benefits for preoperative evaluation and clinical decision-making.

Previous AF recurrence predictive models have incorporated demographic, medical history, electrocardiographic, echocardiographic, and cardiac images (14–16). These predictive models focused solely on clinical parameters, while the pathophysiologic biomarkers were not evaluated. Our predictive model of AF recurrence after catheter ablation incorporates not only the risk factors of atrial structural changes, but also the important biomarkers involved in atrial fibrosis. This type of model is more effective than those using atrial structural changes or biomarkers alone to predict AF. TIMP-1 is involved in the pathophysiological process of atrial fibrosis and can elevate the predictive model accuracy to identify the population with AF recurrence, unlike echocardiology which can be influenced by the examiner. To date, several predictors of recurrence have been identified in various studies. The major risk factors are structural heart disease, LAd, incomplete pulmonary vein (PV) isolation, low LA voltage, C-reactive protein, AF duration, obesity, non-PV triggers, and NT-proBNP (17). In our study, the clinical risk factors were included but the parameters during the catheter ablation procedure were not included. Our model is more suitable for preoperative screening of patients with AF.

The fibrosis biomarker TIMP-1 was incorporated in this model. TIMP-1 plays an important role in the maintenance of extensive atrial fibrosis and AF (18). Enhancement of atrial fibrosis and structural remodeling provides a sufficient substrate for AF. Cardiac fibrosis is a process that leads to an imbalance between extracellular matrix (ECM) deposition and degradation within the heart, resulting in excessive fibroblast proliferation and buildup of ECM proteins within the cardiac interstitial space (19). Increased TIMP-1 activity promotes atrial ECM remodeling in AF (20). TIMP-1 is a tissue inhibitor of metalloproteinases, and a glycoprotein with a molecular weight of 28 kDa (21) that is a natural inhibitor of matrix metalloproteinases (MMPs). MMPs are a group of proteases that are involved in extracellular degradation. Tissue inhibitors of MMPs (TIMPs) can inhibit the proteolytic activity of MMPs in the extracellular matrix. TIMPs are important regulators of ECM renewal, tissue remodeling, and cell behavior (22). An imbalance of activity between TIMPs and MMPs can lead to the degeneration and replacement of the extracellular matrix, leading to atrial remodeling and fibrosis (22, 23). Atrial fibrosis can lead to changes in the refractory period of atrial myocytes, resulting in multiple microreentrants in the atrium and a higher recurrence rate of catheter ablation (24). In patients with advanced atrial fibrosis, AF ablation is associated with a high procedural failure rate (25). Therefore, TIMP-1 levels can reflect the degree of fibrosis and can predict AF recurrence after catheter ablation.

In addition, AF is associated with atrial structural and electrical remodeling. Atrial structural remodeling is evidenced by interstitial fibrosis, leading to atrial dilatation, which in turn reduces the effectiveness of catheter ablation for AF. This increased space between cardiomyocytes, which is likely to occur due to the loss of cells and fibrotic replacement and expansion of ECM, may also cause electrical conduction delays between cardiomyocytes and allow for alternate pathways of conduction (19), such as unidirectional conduction block, slowing of conduction velocity, non-uniform anisotropic propagation, and refractoriness dispersion (26). Voltage reduction in the LA is a diffuse process associated with fibrosis (27). LA voltage in patients with high blood TIMP-1 levels is lower than that in patients with low blood TIMP-1 levels (11, 15), indicating that atrial fibrosis is severe (28), and it provides the substrate for AF occurrence and maintenance. LAd is associated with AF recurrence after catheter ablation and is an independent predictor of AF recurrence (28). Therefore, this predictive model that combines TIMP-1 and LAd can accurately predict AF recurrence after catheter ablation.

In summary, this AF recurrence prediction model including the fibrosis biomarker TIMP-1 and structural remodeling factor LAd had good discrimination ability, accuracy, and clinical benefits. Importantly, the combination of TIMP-1 and LAd may be a valuable tool for identifying patients at risk of AF recurrence before catheter ablation.

### Limitations

Our study has several limitations. First, we conducted a prospective cohort study, which may have introduced unavoidable selection bias. To further confirm the efficiency of these factors in evaluating recurrence after AF catheter ablation, a prospective, multicenter randomized controlled trial is required. Our data warrants further confirmation through a larger sample size study to confirm the prognostic value of TIMP-1 combined with clinical factors in identifying patients at high risk of AF recurrence after catheter ablation. Second, these two biomarkers are not heart-specific, and we did not support our findings using atrial tissue biopsy data or coronary sinus sampling. Third, owing to the small sample size, external verification was not conducted. However, internal verification was conducted 1,000 times by bootstrap and 10 times by cross-validation. The developed model has a high accuracy and discrimination ability. Finally, the relationship between TIMP-1 levels and atrial tissue fibrosis in patients with AF was not investigated in the present study. Future studies should attempt to test this predictive model and collect coronary sinus samples.

In patients with AF who underwent catheter ablation, the combination of the biomarker TIMP-1 and cardiac structural remodeling index LAd can better predict AF recurrence. The prediction model for AF recurrence established in this study has good differentiation ability and a good net clinical benefit.

## Data availability statement

The raw data supporting the conclusions of this article will be made available by the authors, without undue reservation.

## Ethics statement

Approval for this study was obtained from the Ethics Committee of our hospital (No. 2022042X). The patients/participants provided their written informed consent to participate in this study. Written informed consent was obtained from the individual(s) for the publication of any potentially identifiable images or data included in this article.

## Author contributions

WS, HL, HW, and QL performed the material preparation, data collection, and analysis. WS wrote the first draft of the manuscript. All authors commented on the previous versions of the manuscript, contributed to the conception and design of this study, read, and approved the final manuscript.
